# PAAPPAS community trial protocol: a randomized study of obesity prevention for adolescents combining school with household intervention

**DOI:** 10.1186/s12889-016-3473-3

**Published:** 2016-08-17

**Authors:** Michele R. Sgambato, Diana B. Cunha, Viviana T. Henriques, Camilla C. P. Estima, Bárbara S. N. Souza, Rosangela A. Pereira, Edna M. Yokoo, Vitor B. Paravidino, Rosely Sichieri

**Affiliations:** 1 Department of Epidemiology, Institute of Social Medicine, State University of Rio de Janeiro, São Francisco Xavier, 524 – 7° andar, Bloco D, Maracanã, CEP: 20550-900 Rio de Janeiro, RJ Brazil; 2 Department of Epidemiology, Fluminense Federal University, Marques de Paraná, 303 – 3° andar, Prédio anexo, Centro -Niterói, CEP: 24030-210 Rio de Janeiro, RJ Brazil; 3 Department of Social and Applied Nutrition, Federal University of Rio de Janeiro, Av. Carlos Chagas Filho, 373 - 2° andar, Bloco J, Cidade Universitária, CEP: 21941-590 Rio de Janeiro, RJ Brazil; 4 Department of Physical Education and Sports, Naval Academy – Brazilian Navy, Avenida Almirante Silvio de Noronha, s/n, Castelo, CEP: 20021-010 Rio de Janeiro, Brazil

**Keywords:** Obesity prevention, Intervention, Adolescents

## Abstract

**Background:**

The prevalence of childhood obesity is increasing at a high rate in Brazil, making prevention a health priority. Schools are the central focus of interventions aiming the prevention and treatment of childhood obesity, however, randomized trials and cohort studies have not yet provided clear evidence of strategies to reduce prevalence of obesity.

The aim of this study is to present a protocol to evaluate the efficacy of combining school and household level interventions to reduce excessive weight gain among students.

**Methods:**

The intervention target fifth and sixth graders from 18 public schools (9 interventions and 9 controls) in the municipality of Duque de Caxias, metropolitan area of Rio de Janeiro, Brazil. A sample size of 2500 students will be evaluated at school for their weight status and those from the intervention group who are overweight or obese will be followed monthly at home by community health agents. Demographic, socioeconomic, anthropometric, eating behavior and food consumption data will be collected at school using a standardized questionnaire programmed in personal digital assistant. At school, all students from the intervention group will be encouraged to change eating habits and food consumption and to increase physical activity and reducing sedentary behavior.

**Discussion:**

This study will provide evidence whether integration of school with primary health care can prevent excessive weight gain among adolescents. Positive results will inform a sustainable strategy to be disseminated in the health care system in Brazil.

**Trial registration:**

ClinicalTrials.gov, NCT02711488. Date of registration: March 11, 2016.

## Background

The increasing prevalence of obesity in low and middle-income countries [1] requires tailored well-tested preventive strategies to curb the obesity epidemic. In Brazil, children and adolescents had the highest increase in the prevalence of obesity in the last decade [2], particularly in urban low-income areas, which concentrates high percentage of the Brazilian population [3, 4].

Usually, schools are the central focus of interventions aiming prevention and treatment of childhood obesity [5], however, randomized trials and cohort studies have not yet provided clear evidence of strategies to reduce the prevalence of obesity, according to a position paper of the of the U.S. Academy of Nutrition and Dietetics [5]. Potential limitations in these studies include heterogeneity of participants in cluster-randomized trials, baseline imbalance, underestimated sample size and overestimation of possible changes in body mass index [6].

We have been studying prevention of obesity in schools for many years and our studies published from 2008 to 2013 [7–10] have tested various activities for primary prevention. The PAAPPAS study, which stands for “parents, health care agents, students, and teachers for healthy eating” is a school-based randomized research project in its fourth version. In the first initiative, the main intervention focused on the reduction of sodas [7]. The second study was conducted among school cooks [8, 9] with the main aim of reducing use of sugar at school and also to reduce sugar intake and increase physical exercise among the school cooks. The third study, focused on positive messages to increase the intake of water, fruit, and beans and to reduce intake of cookies, sugar-sweetened beverages, and savory snacks [10]. The results of these studies, as well as, many the school-based initiatives based on primary prevention have been promising regarding the modification of behaviors associated with weight gain, such as changing food consumption patterns or physical activity habits, but the impact of these changes in reducing excessive weight gain has not been satisfactory.

Limitations of the primary prevention interventions in their ability to reduce excessive weight gain, despite it efficacy in changing behaviors associated with obesity, could be circumvent by combining preventive school activities with family support for those adolescents obese or in risk of obesity. However, to our knowledge there is no study that had tested combining primary prevention at school with secondary prevention. Brazil is internationally recognized for its development of primary health care at the local level. The Family Health Strategy (FHS) Program in Brazil is a model of assistance centered on health teams, which had improved health outcomes in Brazil [11]. As majority of adolescents in Brazil are students from public schools located in areas covered by FHS, it is reasonable to combine primary and secondary prevention in school-based interventions in order to increase the chances of effectiveness in this context.

Thus, the current study presents the rationale and protocol development, pre-tests, and implementation of the trial PAAPPAS, which combine primary prevention activities at school level with secondary prevention at household level to prevent excessive weight gain.

## Methods

### Study design

The trial is a randomized community controlled trial labeled PAAPPAS, which stands for “parents, students, *community health agents* and teachers for healthy eating”. The intervention aimed at reducing excessive weight gain among adolescents combining primary prevention at schools with the primary care health system, through the FHS Program.

Intervention will occur during the school year of 2016 and target fifth and sixth graders from 18 public schools (9 interventions and 9 controls) in the municipality of Duque de Caxias, metropolitan area of Rio de Janeiro, Brazil.

Primary intervention in the school by trained teachers will provide the overall basis for a healthy lifestyle, including food intake, physical activity and sedentary habits. For adolescents diagnosed with excessive weight, household activities will provide additional motivation to change these behaviors. Trained community health agents (CHA) from the FHS Program will visit the families of adolescents with overweight or obesity every month, integrating activities with adolescent and his/her family.

The study will be conducted according to the Consolidated Standards of Reporting Trials (CONSORT) guidelines for cluster-randomized trials [12]. The Ethics Committee of the Institute of Social Medicine (State University of Rio de Janeiro, Brazil) approved the protocol. Written informed consent was obtained from all the participants’ parents.

### Sample size calculation

The sample size for the secondary prevention intervention was estimated based on a standard deviation of body mass index (BMI) equals 3.0; the difference from baseline to the end of intervention among those with overweight and obesity of 1.1 BMI units and the intracluster correlation coefficient was 0.02, according to a previous study conducted in schools [7]. Considering 80 % power and a 5 % significance level, 117 are needed per group to compare intervention with control. In order to reach this sample assuming a prevalence of overweight/obesity of 20 %, the total sample size needed at school is of 2340 participants. Given an intracluster correlation of 0.02, a 2500 sample size was the goal.

### Setting and participants

**Fig. 1:**
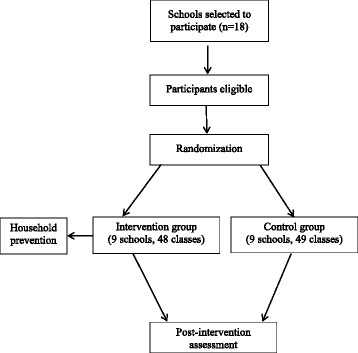
Study design

This study takes place in the municipality of Duque de Caxias (population, 842,686) [13], located 27 km from the state capital and is part of the metropolitan area of Rio de Janeiro. It is one of the poorest areas in the state of Rio de Janeiro, and the prevalence of obesity in this region is rapidly increasing among adults and adolescents [3, 4]. A population based study carried out in Duque de Caxias revealed a mild family food insecurity prevalence of 36 %, and a prevalence of overweight/obesity among adolescents of 24 % [3]. For this study, two of four districts in Duque de Caxias were included, and from 45 municipal schools, 18 schools with fifth and sixth grades classes were selected. All students enrolled in the 5th and 6th grades of selected schools are eligible for this study. The exclusion criteria are students with physical disabilities and pregnant adolescents (Fig. 1).

Schools were randomized half in the control group and half in the intervention group based on number of students using opaque envelopes, in the presence of investigators not involved in the current study. Schools will remain in their allocated group for the duration of the study. Participants in the control arm will receive only the routine activities for healthy behaviour of the school.

### Intervention

The primary prevention at school was based on literature review of eating behaviors, environmental factors associated with obesity [7] and pilot studies. The foods targets were the most important contributors to energy intake identified in the more recent Brazilian survey [14]. The intervention focus on encouraging students to change eating habits and food consumption and encouraging them to increase physical activity and reducing sedentary behavior. Teachers are trained, and every month they receive material for 1-h session of in-class activities (Table 1), except for the first one when students prepare salads. To reinforce the in-class messages, booklets, recipes, and flyers are sent to the families.

**Table 1:** Intervention components, description, dose and expected behavior change

Intervention components	Description	Dose per month	Expected behavior change
School			
1) Healthy eating	Definition of healthy eating and food choices using two educational games.	2 × 30 min	To improve the choices when eating and purchasing foods at supermarkets and fairs.
	Main message: reduction of cookies, sodas, and sugar-sweetened beverages.		
2) Culinary classes	First activity- students assemble salads at school choosing from a large variety of greens and fruits. In the second, they prepare vegetarian pizzas.	1 × 50 min	To develop cooking skills increasing healthy eating choices, to discuss sensory aspects of food and to stimulate teamwork.
3) Water exchange with sugar sweetened beverage	Measuring the amount of sugar in different drinks. Sugar intake and prevention of diseases.	2 × 30 min	To increase water consumption.
	A squeeze with the PAAPPAS logo will be provide to the students and teachers.		To develop a critical view about the consumption of sugar sweetened beverages.
4) Physical activity and sedentary behavior	How many Olympic games they have heard of. What exercises they do and would like to do. Class games related to the Olympic games and time spend sedentary.	2 × 30 min	To stimulate exercise.
5) Fruit consumption	Group discussion and games about of fruits. What they like and dislike. Diversity of fruits from Brazil. How buy fruit al affordable prices and the importance of eating fruits.	2 × 30 min	To exchange cookies consumption by fruit.
6) Mindful eating and self-control strategies	Activity regarding hunger and satiety cues; how to combine foods to make a healthy dishes.	2 × 30 min	To develop eating awareness regarding hunger and satiety physiological signs. To facilitate choices of healthy dishes.
Household	CHA activities		
1) Health eating habits and anthropometry	Measure weight and waist of the adolescent and mother.	30 min	To improve eating habits.
	Encourage adolescents and family to: 1) Keep regular mealtimes; 2) Eat with the family at the table; 3) No television, computer or mobile phone during meals; 4) Eating meal slowly (about 20 min); 5) Serve the dish once, except for vegetables.		
2) Reduce soft drinks and sugar sweetened juices	Show pictures of the amount of sugar in sodas and popular drinks negotiating reduction strategies: 1) Avoiding buying; 2) Replace soft drinks and juices by water and flavored waters; 3) Facilitate adolescent access to fruits; 4) Buy season fruits.	20 min	To reduce soft drinks and sweetened juices and prompt specific strategies to achieve this target.
3) Physical activity and sedentary behavior	Discuss that lifestyle modification are strong allies to lose weight. Provide a list of facilities free of charge in the neighborhood with physical activities such as churches and NGOs. Sedentary reduction strategies: 1) Reduce one hour time in computer games and TV; 2) Encourage the use of physical activity mobile app. 3) Encourage standing or walk during the interval of TV programs or in the game phase shift.	20 min	To increase physical activity and reduce sedentary behavior.
4) Biscuits and sweets	How to read nutritional labels using as example cookies, candy and other high sugar products. Negotiate: 1) Reducing intake of cookies, biscuits and sweets and also avoiding buying them; 2) Replace cookies with homemade popcorn, fresh coconut; peanuts and corn; 3) Substitute processed sweets, with homemade marmalades and jams low in sugar; 4) Add milk to coffee to reduce sugar.	20 min	To reduce biscuits and sweets consumption and prompt specific strategies to achieve this target.
5) Industrialized products	Identify dyes, preservatives and flavor enhance substances in labels avoid buying those high-energy industrialized products. Stimulate: 1) Replacement of noodles; processed sausage and hamburger for homemade burger /ground beef /egg; 2) Clean salads and vegetables in advance for consumption during the week (ask adolescent for help); 3) Keep always in the refrigerator fresh or frozen vegetables and beans; 4) Use spices and make attractive and colorful raw salads and vegetables. 5) Make shopping list to avoid buying unnecessary foods.	20 min	Provide information to reduce the consumption of industrialized products and prompt specific strategies to achieve this target.

Activities for secondary prevention at households, as indicated in Table 1, were based on Paulo Freire’s theory [15]. Secondary prevention is coordinated by the Health Secretary of the Municipality of Duque de Caxias and will be carried out from June to December by the CHA. The CHA will encourage lifestyle changes at family level. The goals are the same as those of the primary prevention with emphasis on eating behaviors and food consumption (reducing soda and sugar sweetened beverages, cookies, sweets, and processed food and increasing fresh foods) and on stimulating the increasing of physical activity and reducing sedentary habits. It also includes clues to reduce total energy intake by families. Each month the adolescent and his/her family are stimulated to choose one of the five goals (Items 1 to 5 in Table 1).

This is the first time that CHA are trained to address obesity at the family level. Goals and all material used in the intervention were developed in partnership with Health and Education authorities in the municipality of Duque de Caxias (Rio de Janeiro, Brazil).

### Outcomes

At the primary prevention level we are testing a reduction of 0.4 units of mean BMI and for the secondary prevention program a reduction of 1.1 units of BMI is expected. Secondary outcomes include body fat and waist circumference. Many behavior changes evaluated will allow measure the adherence to the intervention.

### Data collection at school

Anthropometric measures, food consumption and a questionnaire are being collected at baseline [March-April] and post-intervention [November-December] using personal digital assistant (PDA). Students will complete a structured questionnaire under the supervision of field researchers using PDA and the CHA will take anthropometric measurements.

Food consumption will be assessed by 24-h recall and a short (23 items) food frequency questionnaire (FFQ), which is a reduced version of an FFQ validated for adolescents of Rio de Janeiro [16]. The 24-h recall will use the BrasilNutri software, entering data during interviews in schools. The software encompasses a computerized food database developed for the 2008-2009 nationwide dietary survey [17].

Measurement of physical activity will include the evaluation of exercise, leisure activities, commuting to school, and sedentary activities (video games, television and computer time) measured by questionnaire [18] and in a subgroup by accelerometer (GT3X Actigraph).

### Anthropometric and body composition

Weight, height and waist circumference at the lower value were measured using standardized procedures [19] and body composition estimated by bioelectrical impedance, using the leg-to-leg Tanita scale (BC-558). Height was measured using a portable stadiometer. Overweight and obesity classification of the World Health Organization was used [20].

### Statistical analysis

Intent to treat analysis will be conducted through mixed models longitudinal analysis taking into account the cluster effect (classes) [21]. These mixed models allow for the exploration of factors associated with BMI variation and the other outcomes in a multilevel analysis where the class was on the second level and the individual measures on the first level. Data analysis will be performed using the Statistical Analysis System, version 9.3 (SAS Institute Inc, Cary, NC).

## Discussion

This is the first study that integrates school intervention with primary health care to prevent excessive weight gain among adolescents. Our previous studies and most of the literature have shown inconsistent results of studies based on only in the school, thus this is a promising study.

Our first school-based prevention trial was based on exchange sodas by water [7] and was inspired in a United Kingdom study that showed positive results with this approach [22]. In contrast with the British study, our results did not observe reduction in total intake of sugar; even though positive behavior changes were verified, adolescents increased the intake of sugary fruit-based drinks in replacement of sodas. The study coincided with the moment when food industry boosted the market of processed fruit-based drinks, which have as much or higher amount of sugar than sodas [7]. Therefore, in the second version of the study [10] we targeted the most important contributors of sugar intake in Brazil (all sugar sweetened beverages, sweets, cookies) [23] and behavior modifications were achieved, but without change in the weight gain.

In 2014, we tested a secondary prevention intervention of obesity in one school, mainly focusing on increasing physical activity of overweight/obese students and inviting their families to come to school to discuss possible strategies for changing lifestyle. However, family participation was minimal and overweight or obese adolescents did not enrolled in the after-school physical activity classes (unpublished results). The present study includes the positive results in changing behavior from previous ones and emphasis an interaction with primary health care based on family strategy.

We expect that FHS will facilitate family participation, which is very low in low-income communities. All families to be included in the study live in catchment areas of the FHS units allowing for this innovative approach combining primary and secondary prevention to address the escalating public health problem of obesity in Brazil.

The CHA act in the territory in which she/he is affiliated and serve as a mediator between families and health professionals. The family within the community is an active subject in the care process. This involves considering the family’s background and empowering families to face the health problems of their members. Thus, the FHS model integrates biomedical-institutionalized knowledge with the voice of the community [24]. More recently, nutritionists were incorporated in the FHS and guidelines for diabetes and hypertension have been developed [11], but there are no established guidelines for childhood obesity. Thus, this project will inform possible strategies to be disseminated in the health care system in Brazil.

The results of this study will be submitted for publication in next year and the methods presented may contribute to develop effective interventions to reduce the growing prevalence of obesity. If positive results of the CHA intervention are observed, all CHA from Duque de Caxias, Rio de Janeiro will be trained and materials developed used in the primary health care.

## Abbreviations

CHA, community health agents; CONSORT, consolidated standards of reporting trials; FFQ, food frequency questionnaire; FHS, family health strategy; PAAPPAS, parents, students, *community health agents* and teachers for healthy eating; PDA, personal digital assistant
